# Antagonistic effects of IL-17 and Astragaloside IV on cortical neurogenesis and cognitive behavior after stroke in adult mice through Akt/GSK-3β pathway

**DOI:** 10.1038/s41420-020-00298-8

**Published:** 2020-08-10

**Authors:** Li Sun, Ruili Han, Fei Guo, Hai Chen, Wen Wang, Zhiyang Chen, Wei Liu, Xude Sun, Changjun Gao

**Affiliations:** 1 Department of Anesthesiology, The Second Affiliated Hospital of Air Force Medical University, 710038 Xi’an, Shaanxi Province China; 2 School of Basic Medicine, Air Force Medical University, 710032 Xi’an, Shaanxi Province China

**Keywords:** Attention, Apoptosis, Neural stem cells, Adult neurogenesis, Neuroimmunology

## Abstract

We aimed to investigate the exact effect of IL-17 on regulating neural stem cells (NSCs) stemness and adult neurogenesis in ischemic cortex after stroke, how Astragaloside IV(As-IV) regulated IL-17 expression and the underlying mechanism. Photochemical brain ischemia model was established and IL-17 protein expression was observed at different time after stroke in WT mice. At 3 days after stroke, when IL-17 expression peaked, IL-17 knock out (KO) mice were used to observe cell proliferation and neurogenesis in ischemic cortex. Then, As-IV was administered intravenously to assess cell apoptosis, proliferation, neurogenesis, and cognitive deficits by immunochemistry staining, western blots, and animal behavior tests in WT mice. Furthermore, IL-17 KO mice and As-IV were used simultaneously to evaluate the mechanism of cell apoptosis and proliferation after stroke in vivo. Besides, in vitro, As-IV and recombinant mouse IL-17A was administered, respectively, into NSCs culture, and then their diameters, viable cell proliferation and pathway relevant protein was assessed. The results showed knocking out IL-17 contributed to regulating PI3K/Akt pathway, promoting NSCs proliferation, and neurogenesis after ischemic stroke. Moreover, As-IV treatment helped inhibit neural apoptosis, promote the neurogenesis and eventually relieve mice anxiety after stroke. Unsurprisingly, IL-17 protein expression could be downregulated by As-IV in vivo and in vitro and they exerted antagonistic effect on neurogenesis by regulating Akt/GSK-3β pathway, with significant regulation for apoptosis. In conclusion, IL-17 exerts negative effect on promoting NSCs proliferation, neurogenesis and cognitive deficits after ischemic stroke, which could be reversed by As-IV.

## Introduction

Stroke is defined as a leading cause of adult disability, due to its high morbidity and mortality and long-term psychological^1,2^. It is estimated that 15 million people in the world are affected by stroke every year, 5 million of which die and another 5 million suffer from long-term disability^3^. Thus, further research is demanded to explore the underlying mechanism of ischemic stroke to find more efficient treatment strategy.

Interleukin-17 (IL-17, also called IL-17A), a pro-inflammatory cytokine mainly derived from γδ T cells and T helper 17 (Th17) cells, also has an important role in a wide variety of neurological diseases^4^. It has been found that expression of IL-17 is upregulated by ischemic stroke and the over-expression of IL-17 indicates a poorer treatment effect and prognosis^5–7^. However, IL-17 inhibits autophagy through activating phosphatidylinositol-4,5-bisphosphate 3-kinase (PI3K) pathway, one of the most popular pathways to regulate cell proliferation and apoptosis, to interrupt the Glycogen synthase kinase-3β (GSK-3β)-mediated degradation of BCL2 in lung epithelial cells^8^. Some results also suggest that IL-17 plays an essential role in inhibiting proliferation and differentiation via Wingless/integrated (Wnt) signaling^9–12^. Administrating IL-17A can nullify the microglial M2 polarization to inhibit anti-inflammation activity^13^. Besides, the inhibitory effect of IL-17 on anti-inflammation which can be reversed at the GSK-3β level by PI3K/Akt signaling pathway^14^. All these studies indicate the complex and promising role of IL-17 on cell proliferation and inflammation. Furthermore, IL-17 has a quite complex role in regulating adult neurogenesis. For example, IL-17 negatively regulates adult neurogenesis and proliferation in the dentate gyrus (DG) of adult hippocampus^15,16^. However, IL-17A could also maintain and augment survival and neuronal differentiation of neural progenitor cells (NPCs) in the subventricular zone (SVZ) after ischemic stroke^17^. Speaking of neurogenesis, it is inevitable to refer to GSK-3β, inhibition of which increases the proliferation of neural progenitors and promotes neurogenesis in some research^18–20^. Pro-differentiation factors such as Wnt and pro-proliferation factors secreted by astrocytes can also stimulate neurogenesis^21^. Therefore, whether IL-17 plays a negative role on neurogenesis by regulating cell proliferation and inflammation after stroke and the exact mechanism underlying is still not determined.

Astragaloside IV (As-IV), a primary bioactive compound of Radix Astragali, Astragalus mongholicus Bunge (Fabaceae), has been described as potential neuroprotective effect in experimental models of Alzheimer’s disease and cerebral ischemia^22^. It has been found As-IV protected neurons from apoptosis and autophagy^23,24^, some by regulating Akt/GSK-3β/ β-catenin pathway^25–27^. As-IV could be a new therapeutic drug candidate for post-stroke treatment and effectively promote neural stem cells (NSCs) proliferation and neurogenesis in transient cerebral ischemic brains^28^. In our previous study, As-IV exerts cognitive benefits and promotes hippocampal neurogenesis and synaptic plasticity in stroke mice via Wnt pathway. IL-17 expression is downregulated by administering As-IV^29^, which is consistent with the results in other acute diseases^30–32^. Therefore, it seems that As IV could be a promising strategy in the therapeutic arsenal against ischemic cortex after stroke for its effect on activating proliferation and promoting neurogenesis.

Based on these research, we hypothesize that inflammatory factor IL-17, upregulated by ischemic injury in adult cortex, could regulate cell proliferation, apoptosis and inflammation, and eventually suppress neurogenesis, which can be reserved by administering As-IV, and the mechanism underlying may be involved in Akt/GSK-3β pathway.

## Results

### IL-17 protein and mRNA expression peaks at 3 days, when microglial M2 polarization is inhibited by IL-17

**Fig. 1:**
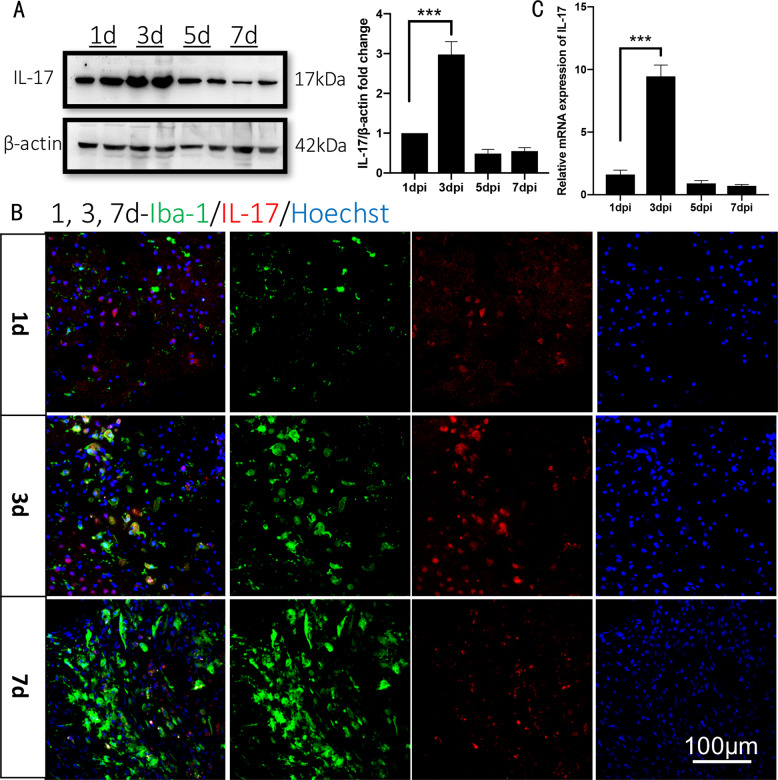
IL-17 expresses differently at different time after stroke and peaks at 3 days in WT mice. **a** IL-17 protein expression in the ischemic cortex of WT mice was measured at 1, 3, 5, and 7 dpi by western blotting. Data represent mean ± SEM, *n* = 3; ***P < 0.001. **b** Double-immunostaining for Iba-1 (green)/IL-17 (red) in the ischemic cortex at 1, 3, and 7 dpi in WT. **c** IL-17 mRNA in the ischemic cortex was measured by real-time RT-PCR for different time. Data represent mean ± SEM, *n* = 3; ***P < 0.001. IL: interleukin; dpi: days post injury.

The temporal expression profile of IL-17 in ischemic cortex after stroke indicates that IL-17 mRNA and protein expression change at different time after stroke^17^. The results in present study showed that IL-17 mRNA and protein expression appeared after stroke and peaked at 3 dpi (*n* = 5, Fig. 1a, c). It is reported that microglial M2 polarization after cerebral injury, identified by elevated Iba-1 protein expression, exhibits anti-inflammation activity, which can be nullified by administrating IL-17A^13^. In our study, Iba-1 protein expression increases after stroke, but the expression peak time was far later than that of IL-17. Iba-1 protein expression did not increase hastily until IL-17 expression had decreased in an early stage, before the third day after the stroke (*n* = 5, Fig. 1b). The relationship that the inhibition of Iba-1 was offset with gradually decreased IL-17 was quite consistent with the previous conclusion that increased Iba-1 protein expression is nullified by administrating IL-17A.

### Differentially expressed mRNA and protein by knocking out IL-17 is enriched in PI3K/Akt pathway

**Fig. 2:**
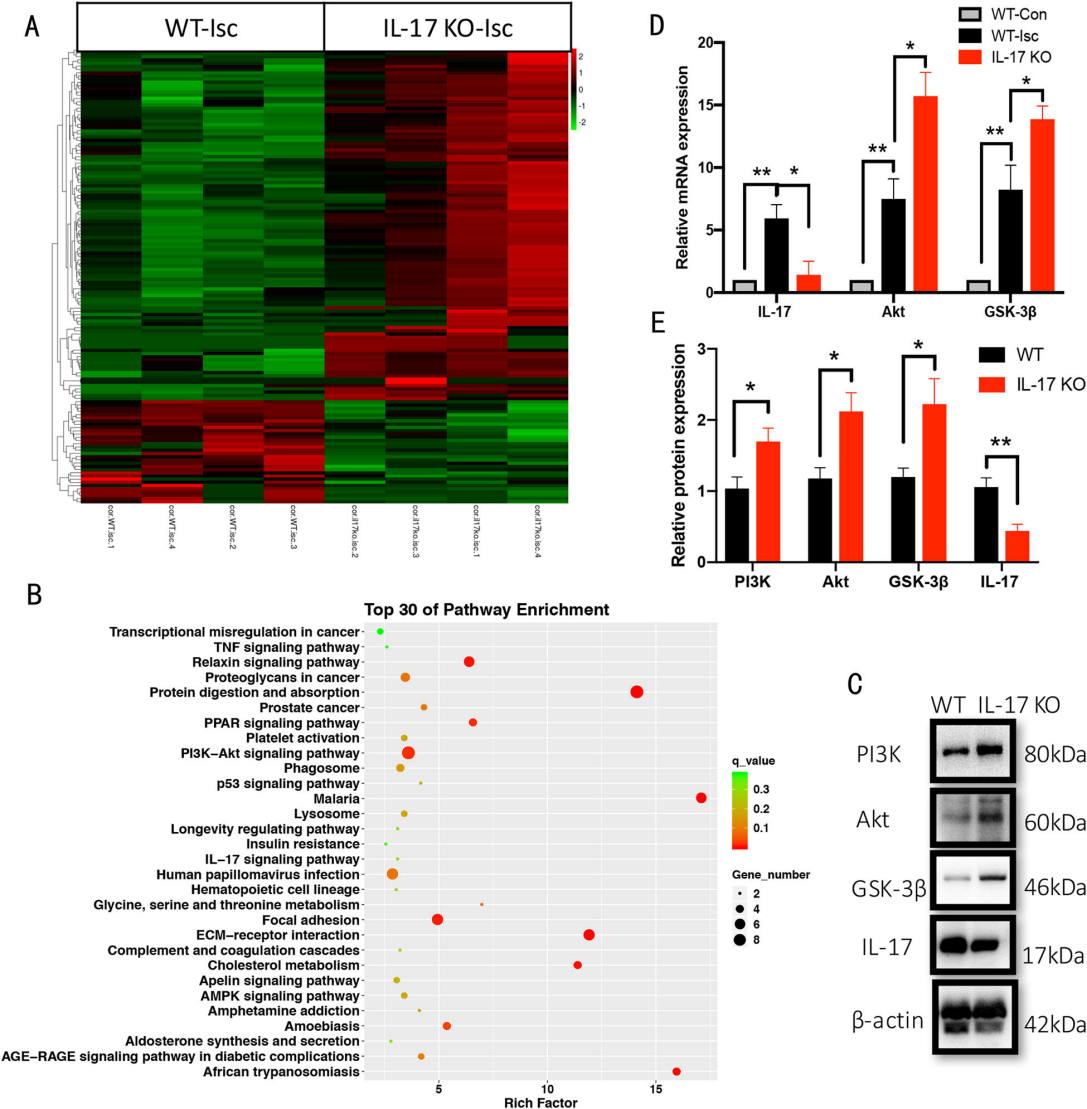
Differentially expressed mRNA and protein by knocking out IL-17 is enriched in PI3K/Akt pathway. **a** Transcriptome profiles from ischemic cortex in WT and IL-17 KO mice showed DEGs, *n* = 4. Bands with red, black or green in the heat map indicated high, moderate or low expression, respectively. **b** The top 30 of pathway enrichment was analyzed (**c**, **e**) PI3K, Akt, GSK-3β, and IL-17 protein expression in ischemic cortex was measured between WT and IL-17 KO mice by western blotting. Notice the increase of PI3K, Akt and GSK-3β and the decrease of IL-17 in ischemic cortex in IL-17 KO mice. Data represent mean ± SEM, *n* = 3; *P < 0.05, **P < 0.01. **d** The real-time RT-PCR was conducted for assessing IL-17, Akt and GSK-3β. Notice the increase of Akt and GSK-3β in IL-17 KO mice. Data represent mean ± SEM, *n* = 3; *P < 0.05, **P < 0.01. IL: interleukin; KO: knock out; WT: wild type; Isc: ischemic cortex; Con: contralateral cortex; DEGs: differentially expressed genes; PI3K: phosphatidylinositol-4,5-bisphosphate 3-kinase; GSK-3β: Glycogen synthase kinase-3β.

Transcriptome sequencing analysis showed that 109 genes are significantly upregulated and 32 genes downregulated by knocking out IL-17 in ischemic cortex (n = 4, Fig. 2a). PI3K/Akt pathway relevant genes, including *Col1a1, Col1a2, Col6a3, Spp1, Nr4a1, Thbs2, Creb3l1*, are increased significantly by knocking out IL-17 in pathway enrichment (Fig. 2b). The expression profile regarding *Il-17, Akt*, and *Gsk-3β* in the areas was further confirmed by real-time RT-PCR (*n* = 5, Fig. 2d). Besides, PI3K, Akt, and GSK-3β protein expression was also increased at 3 dpi by knocking out IL-17, with decreased IL-17 (*n* = 5, Fig. 2c, e).

### NSCs’ stemness and neurogenesis in ischemic cortex is activated by knocking out IL-17, with infarction area increased

**Fig. 3:**
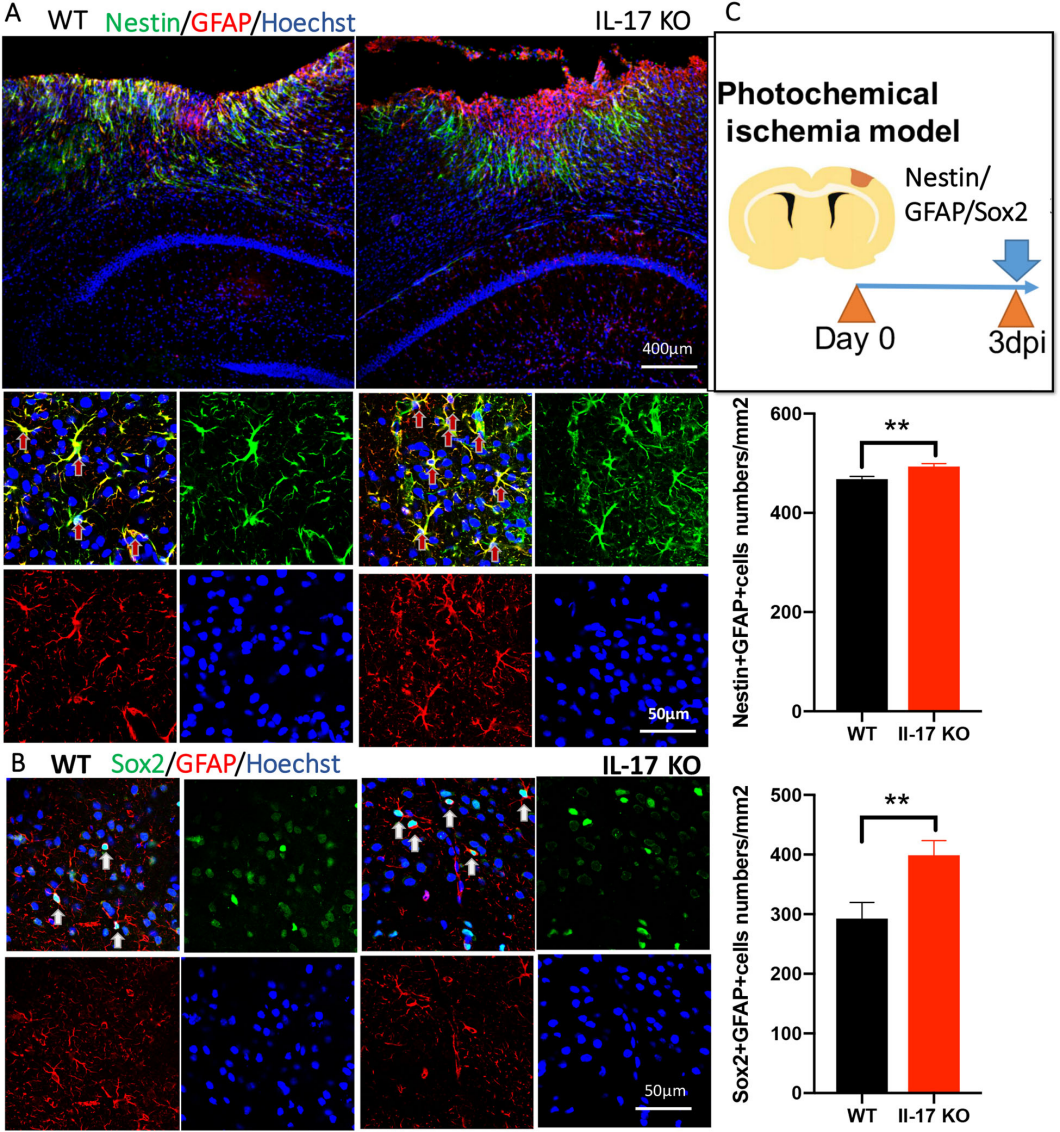
NSCs’ stemness around the ischemic cortex is activated by knocking out IL-17. **a**, **b** Double-immunostaining for Nestin (green)/GFAP (red) and Sox2 (green)/GFAP (red) in the ischemic cortex at 3 dpi in WT and IL-17 KO mice. Double positive cells numbers were analyzed in infarcted area per square millimeter. Data represent mean ± SEM, *n* = 4; **P < 0.01. **c** Experimental scheme for assessing NSCs proliferation in ischemic cortex. WT: wild type; IL: interleukin; KO: knock out; GFAP: glial fibrillary acidic protein; NSCs: neural stem cells.

Quiescent NSCs can be activated by ischemic injury, and Nestin and Sox2 are identified as stem cell markers^33^. Astroglia is the most abundant type of glial cells in the CNS. The expression of astrocyte marker glial fibrillary acidic protein (GFAP) is determined to investigate the astrocytes activity. First, we focused mainly on the activated NSCs in ischemic cortex at 3 dpi in the present study (Fig. 3c). The results showed a significant increase of Nestin^+^, Sox2^+^, and GFAP^+^ cell numbers around the ischemic cortex at 3 dpi, indicating activated quiescent NSCs. IL-17 KO mice had greater double positive cell numbers of Nestin/GFAP and Sox2/GFAP per unit area around the ischemic infarction cortex, compared with WT mice (*n* = 5, Fig. 3a, b**)**. Interestingly, Nestin and GFAP positive cells were highly coincident (Fig. 3a), indicating the activated NSCs around the infarction zone may derive from GFAP^+^ astrocytes.

**Fig. 4:**
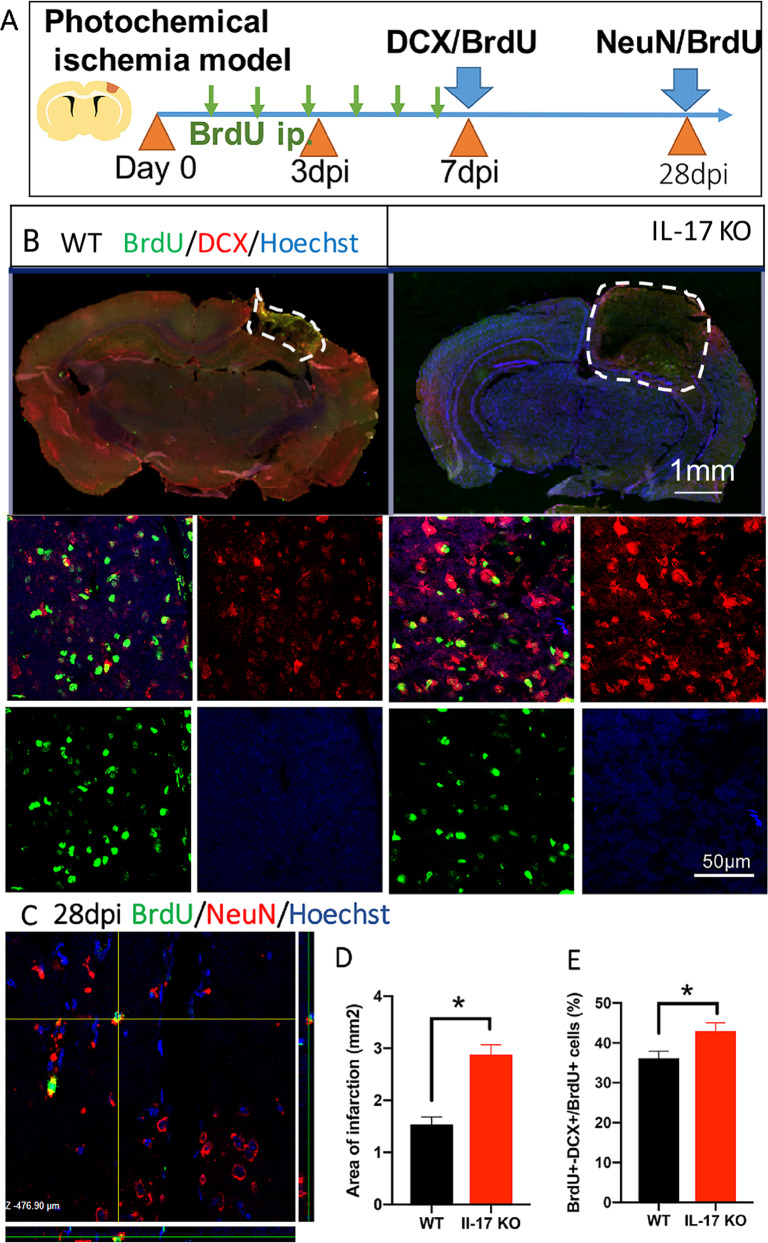
Infarction area and neurogenesis in ischemic cortex is promoted by knocking out IL-17. **a** Experimental scheme for assessing neurogenesis in ischemic cortex. **b** Double-immunostaining of BrdU (green)/DCX (red) and the quantification at 7 dpi after stroke in WT and IL-17 KO mice. **c** Double-immunostaining of BrdU (green)/NeuN (red) at 28 dpi in WT mice cortex. **d**, **e** Notice the increase of infarction area and BrdU^+^-DCX^+^/BrdU^+^ cells percentage in ischemic cortex in IL-17 KO mice. Data represent mean ± SEM, *n* = 4; *P < 0.05. IL: interleukin; KO: knock out; DCX: doublecortin.

Next, to observe the cortical neurogenesis, BrdU was administered intraperitoneally (ip.) for six or 14 consecutive days after stroke **(**Fig. 4a**)**. We evaluated doublecortin (DCX) expression because DCX is a marker for neuronal precursors and DCX^+^ cells are rare within normal adult cortex^34^. However, an increase in DCX immunostaining was readily visible in the ischemic cortex at 7 dpi **(**Fig. 4b**)**, indicating that cerebral ischemia in cortex stimulates neurogenesis. Moreover, adult cortical neurogenesis was detected with BrdU and DCX double marker. Among BrdU^+^ cells, BrdU and DCX double positive cell numbers in IL-17 KO mice were increased significantly, compared with WT mice (*n* = 5, Fig. 4b, e). Significant increasing infarction area was also observed in IL-17 KO mice, compared with WT mice (*n* = 5, Fig. 4d), indicating that IL-17 exerts protective effect on cell death in infarction area. Not all DCX-positive neuroblasts will convert into neurons. So we further performed double immunofluorescent staining with mature neuron-specific marker NeuN and BrdU to detect newborn neurons at 28 dpi in WT mice **(**Fig. 4c**)**. However, the numbers of BrdU^+^-NeuN^+^ cells in IL-17 KO mice were not significantly increased in the ischemic cortex.

### Neurogenesis in ischemic cortex is promoted and anxiety-like behavior is relieved by administering As-IV

**Fig. 5:**
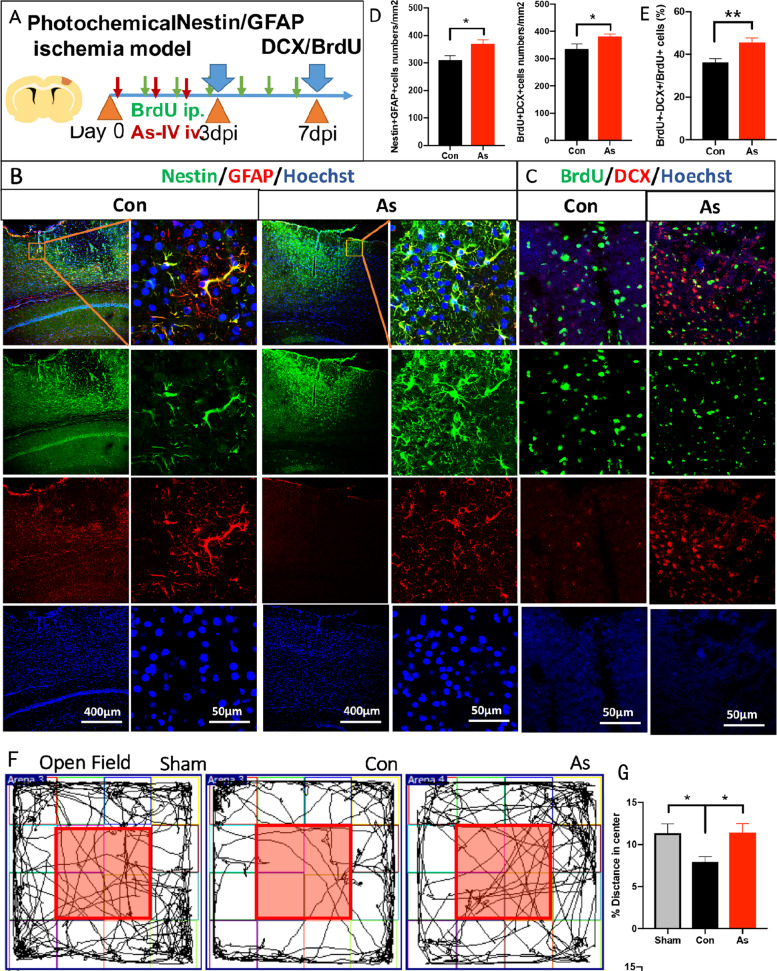

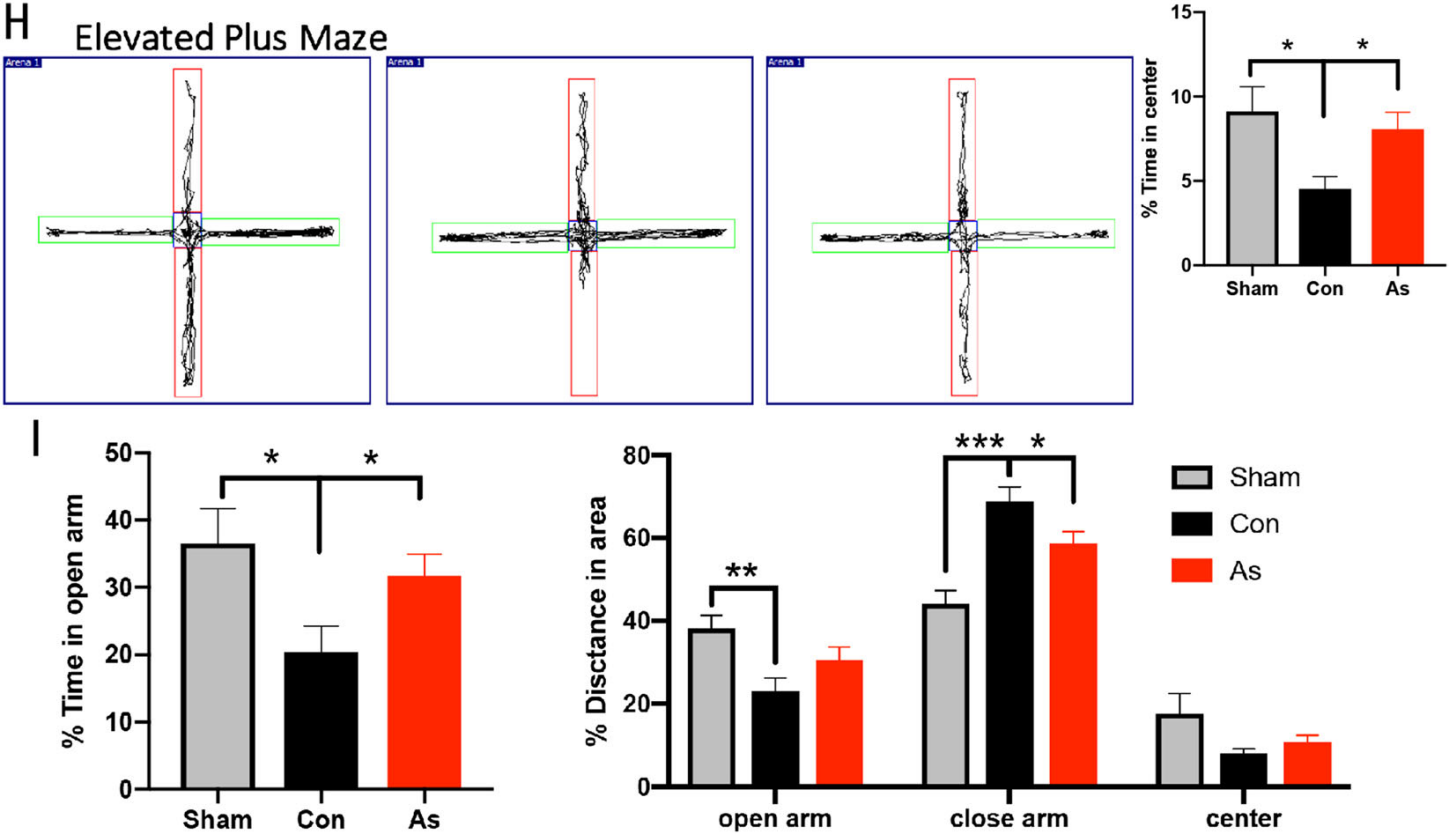
Neurogenesis in ischemic cortex is activated and anxiety-like behavior is relieved by administering As-IV. **a** Experimental scheme for assessing NSCs’ stemness and neurogenesis in ischemic cortex. **b**, **c** Double-immunostaining of Nestin (green)/GFAP (red) at 3 dpi and BrdU (green)/DCX (red) at 7 dpi after stroke. **d** Notice the increase of double positive cells numbers of Nestin^+^-GFAP^+^ in infarcted area per square millimeter. Data represent mean ± SEM, *n* = 3; *P < 0.05. **e** Notice increased double positive cells numbers of BrdU^+^-DCX^+^ and BrdU^+^-DCX^+^/BrdU^+^ cells percentage in As-IV treated mice. Data represent mean ± SEM, *n* = 3; *P < 0.05, **P < 0.01. **f** Open field assay of sham, control and As-IV treated mice. **g** Notice the decrease of time and distance in center area in control group compared with the sham group. The time and distance in center area was increased in As-IV treated mice, compared with the control group. Data represent mean ± SEM, *n* = 7; *P < 0.05. **h** Elevated plus maze test of sham, control and As-IV treated mice. **i** Notice the decrease of time and distance in the open field and open arm in control group compared with the sham group. The time and distance in the open field and open arm was increased in As-IV treated mice, compared with the control group. Data represent mean ± SEM, *n* = 7; *P < 0.05, ***P < 0.001. As: Astragaloside IV; Con: control; GFAP: glial fibrillary acidic protein; DCX: doublecortin.

To observe the activation of NSCs around the ischemic cortex and neurogenesis in the ischemic cortex, Nestin/GFAP at 3 dpi and DCX/BrdU at 7 dpi immunohistochemistry staining was compared between control WT mice and As-IV treated WT mice (Fig. 5a). Mice treated with As-IV had greater double positive cell numbers of Nestin/GFAP in the ischemic infarction cortex compared with mice without administering As-IV (*n* = 5, Fig. 5b–d). Double positive cell numbers of DCX/BrdU and double positive cells/BrdU^+^ cells percentage was increased by giving As-IV **(***n* = 5, Fig. 5e**)**. We next investigated whether As-IV could release anxiety in mice. At 3 dpi, in comparison with sham mice, ischemic stroke mice showed significant reduction of moving distance and time spent in the center of open field, while As-IV reversed the reduction for moving distance and time spent (*n* = 7, Fig. 5f, g). In elevated plus maze test, the moving time and distance spent in the open arm of ischemic stroke mice was significantly decreased for stroke mice, compared with sham mice, while As-IV reversed the reduction (*n* = 7, Fig. 5h, i). These data demonstrated that the anxiety-like behavior appeared following ischemic stroke and relieved by administering As-IV.

### Neural apoptosis in ischemic cortex is ameliorated by administering As-IV, with PI3K/Akt pathway upregulated

**Fig. 6:**
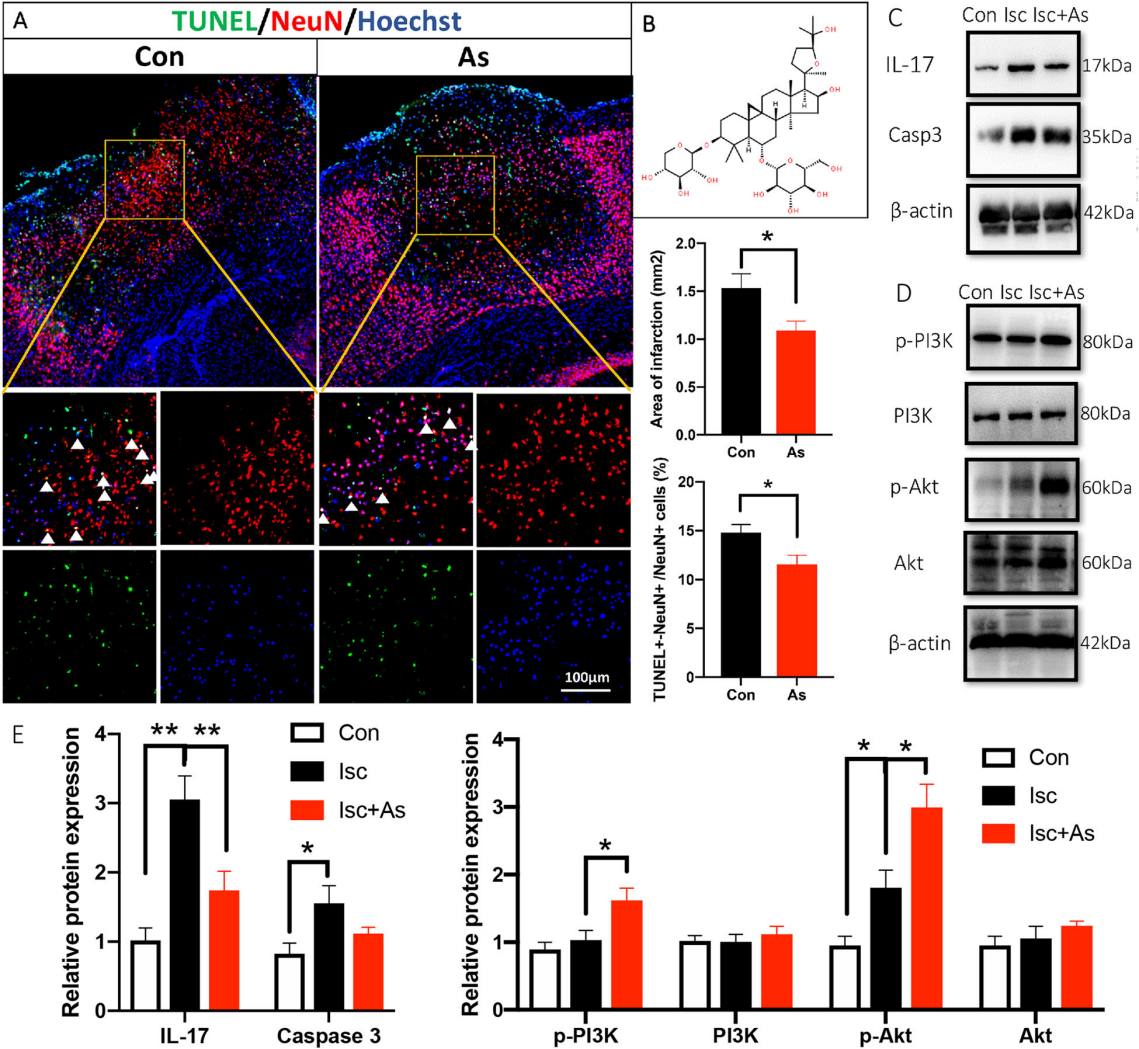
Area of infarction and neural apoptosis in ischemic cortex is ameliorated by administering As-IV, with PI3K/Akt pathway upregulated. **a** Double-immunostaining of TUNEL (green)/NeuN (red) at 3 dpi after stroke. Notice the decrease of infarction area and TUNEL^+^-NeuN^+^/NeuN^+^ cells in ischemic cortex in As-IV treated mice. Data represent mean ± SEM, *n* = 4; *P < 0.05. **b** As-IV structure. **c**–**e** Western blotting and quantitative data for IL-17, Caspase 3, p-PI3K, PI3K, p-Akt, and Akt in the contralateral and ischemic cortex in WT mice, with treating As-IV at 3 dpi. Data represent mean ± SEM, *n* = 4; *P < 0.05, **P < 0.01. TUNEL: TdT mediated dUTP nick end labeling; As: Astragaloside IV; Con: control.

As-IV is a primary bioactive compound of Radix Astragali **(**Fig. 6b**)**, which exerts beneficial effect on cognitive recovery after stroke^23^. Further analysis in our study showed that, at 3 dpi, there was significantly less infraction area and less double positive TUNEL and NeuN cells numbers/NeuN positive cells numbers percentage in the cortex of As-IV treated mice (*n* = 5, Fig. 6a). Previous studies have suggested that As-IV inhibits IL-17 expression^30,32^ and pro-apoptotic protein Caspase 3 expression^35^ in other animal models. In this study, we first observed how As-IV made effect on IL-17 and Caspase 3. The results showed that As-IV did downregulate the expression of IL-17 and Caspase 3 elevated by ischemic injury in cortex at 3 dpi (*n* = 5, Fig. 6c). As-IV treatment resulted in a profound decrease in the phosphorylated forms of Akt and GSK-3β, according to some research^27^. Consistently, in this study, the expression of p-PI3K and p-Akt was upregulated by As-IV treatment in ischemic cortex, but no significant difference of the total PI3K and Akt protein expression was found (*n* = 5, Fig. 6d). Therefore, As-IV not only exerts anti-apoptosis effect, with decreasing infarction area, TUNEL positive cells numbers and Caspase 3 protein expression, but also pro-proliferation effect, with upregulating p-PI3K and p-Akt.

### IL-17 is a key effector for As-IV to regulate cell apoptosis and proliferation, with upregulating Wnt/β-catenin pathway

**Fig. 7:**
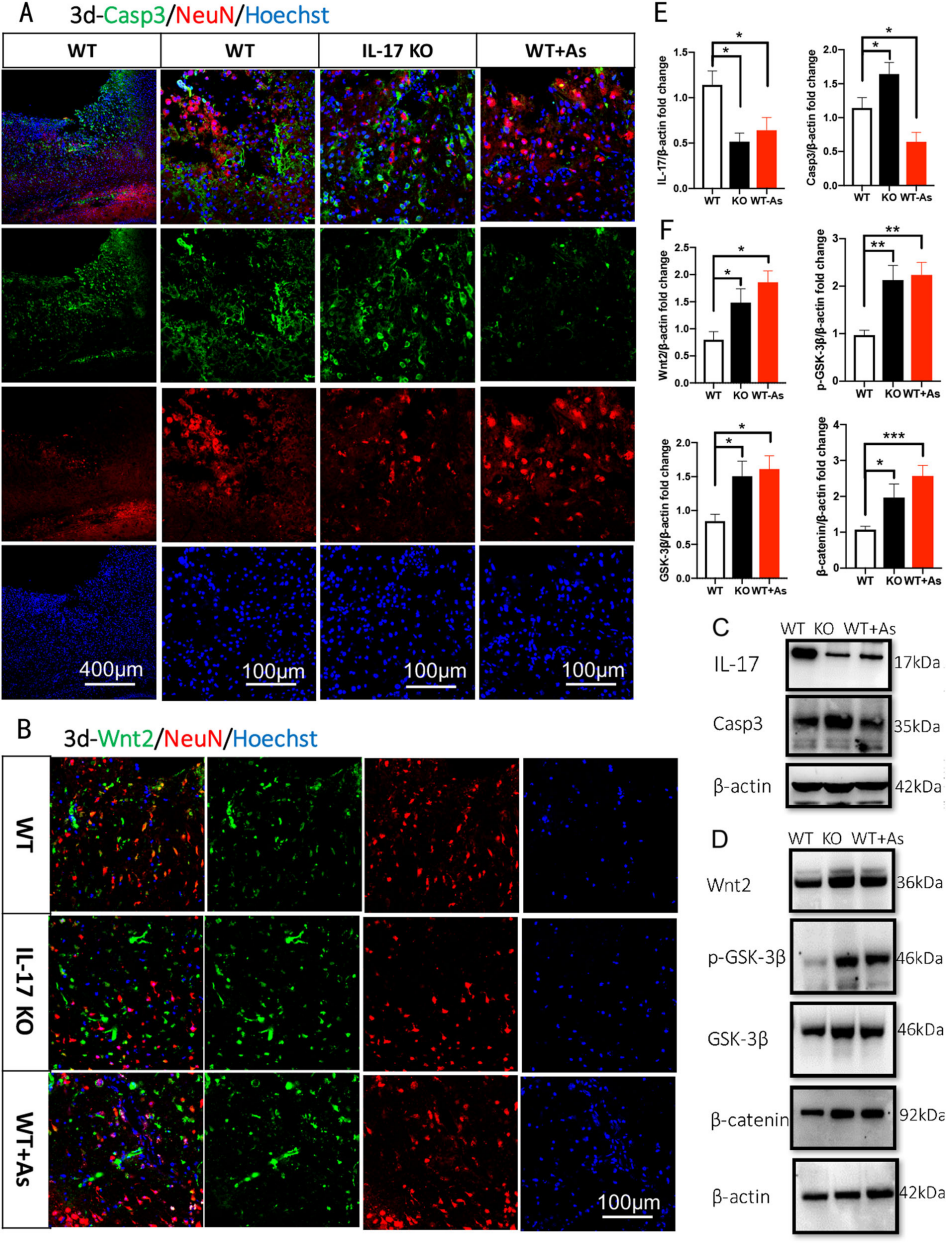
Antagonistic effects of IL-17 and As-IV on regulating cell inflammation, apoptosis and proliferation. **a**, **b** Double-immunostaining of Caspase 3 (green)/NeuN (red) and Wnt2 (green)/NeuN (red) at 3 dpi after stroke. **c**, **e** Western blotting and quantitative data for IL-17 and Caspase 3 in the ischemic cortex of WT, IL-17 KO and As-IV treated WT mice at 3 dpi. Data represent mean ± SEM, *n* = 4; *P < 0.05. **d**, **f** Western blotting and quantitative data for Wnt2, p-GSK-3β, GSK-3β and β-catenin of WT, IL-17 KO and As-IV treated WT mice at 3 dpi. Data represent mean ± SEM, *n* = 4; *P < 0.05, **P < 0.01, ***P < 0.001. As: Astragaloside IV; IL: interleukin; KO: knock out; WT: wild type; Isc: ischemic cortex; Con: contralateral cortex; PI3K: phosphatidylinositol-4,5-bisphosphate 3-kinase; GSK-3β: Glycogen synthas kinase 3β.

Firstly, IL-17 was downregulated significantly by giving As-IV (Fig. 7c, e). Members of the Caspase family of proteases play essential roles in the initiation (Caspase-2, -8, -9 and -10) and execution of apoptosis (Caspase-3, -6, and -7)^36^. We found that Caspase 3 expression was increased by knocking out IL-17, and decreased by giving As-IV (*n* = 5, Fig. 7a, c, e), which conformed the opposite effect of knocking out IL-17 and giving As-IV working on cell apoptosis. Furthermore, Wnt/β-catenin signaling pathway plays an important role in early embryonic development, organ formation, and tissue regeneration, which can be activated by As-IV^37,38^. So, in the present study, Wnt2, GSK-3β and β-catenin protein expression was observed for neural regeneration. We found that such protein expression was upregulated by both knocking out IL-17 and administering As-IV (*n* = 5, Fig. 7d, f). Wnt 2 positive cells and NeuN positive cells were observed in the core of the ischemic cortex, and the Wnt2^+^ cells numbers were increased by both knocking out IL-17 and giving As-IV. We found that there were only few double positive cells observed in ischemic core (Fig. 7b), suggesting that Wnt2 protein derive from other type of cells, not neurons, in an early stage of ischemia.

### In vitro, NSC proliferation ability is promoted by administering As-IV but inhibited by IL-17A

**Fig. 8:**
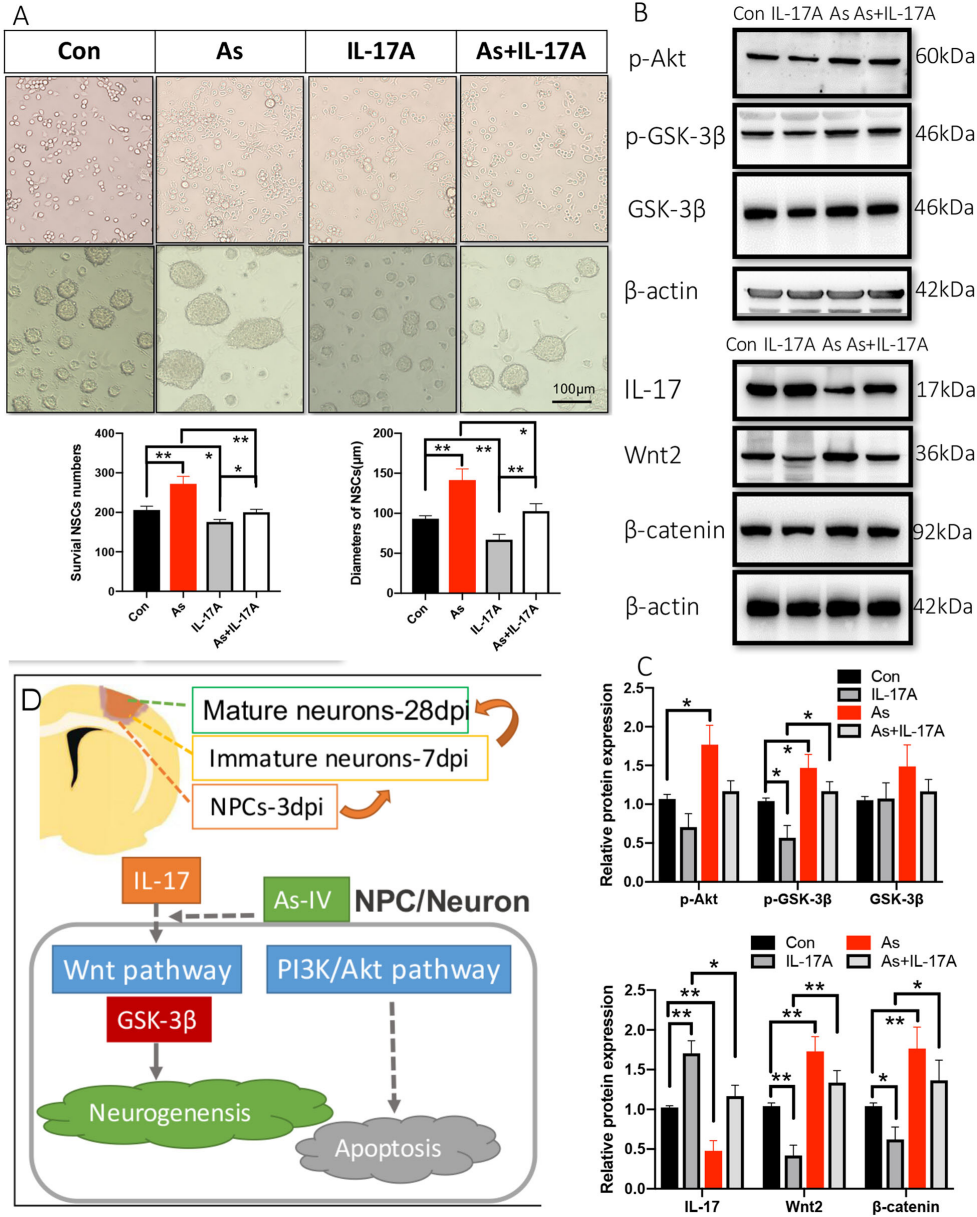
Neurospheres’ diameter and NSCs’ proliferation ability is promoted by As-IV but inhibited by IL-17A in vitro. **a** Proliferation ability of viable NSCs in culture by treating PBS, As-IV and IL-17A respectively was analyzed. Notice the increase of NSCs’ diameter and proliferation ability in As-IV treated culture and decrease in IL-17A treated culture. Data represent mean ± SEM, results are representative of three independent experiments, *P < 0.05, **P < 0.01. **b** Western blotting and quantitative data for p-Akt, p-GSK-3β, GSK-3β, IL-17, Wnt2, and β-catenin in different culture treated PBS, IL-17A, As-IV and IL-17A + As-IV, respectively. **c** Notice the increased expression of p-Akt, p-GSK-3β, Wnt2, and β-catenin by treating As-IV, with IL-17 expression decreased. Notice the decreased expression of p-Akt, p-GSK-3β, Wnt2, and β-catenin by treating As-IV, with IL-17 expression increased. Significant increase of p-Akt, p-GSK-3β, Wnt2, and β-catenin protein expression was observed in IL-17A treated culture, with decreased IL-17. Data represent mean ± SEM, *P < 0.05, **P < 0.01. **d** Schematic drawing of the experimental design. As: Astragaloside IV; Con: control; IL: interleukin; PI3K: phosphatidylinositol-4,5-bisphosphate 3-kinase; GSK-3β: glycogen synthase kinase 3β; NPCs: neural precursor cells.

Defining the mechanism that regulates NSC fate is critical to increase our understanding of neurogenesis. NSCs are capable of self-renewal and regeneration and we first determined the effect of 20 μM As-IV and 1 ng/ml recombinant mouse IL-17A on sphere formation and adherent culture cells proliferation, a well-accepted biological property representing stemness. The results showed that stemness was activated by treating As-IV and inhibited by giving IL-17A (*n* = 5, Fig. 8a). To understand the underlying mechanism of regulating cultured NSCs’ stemness, Akt/GSK-3β pathway and Wnt/β-catenin pathway protein expression was observed by western blots. The results showed that p-Akt, p-GSK-3β, Wnt2, and β-catenin protein expression was decreased by administering exogenous IL-17A, and the inhibition of the NSC proliferation by IL-17A was offset by administering As-IV (*n* = 5, Fig. 8b, c). Unsurprisingly, IL-17 protein expression was increased by giving IL-17A and decreased by giving As-IV, which indicated the inhibitory effect of As-IV on IL-17 expression.

## Discussion

Stroke remains a leading cause of adult disability and the demand for therapy is growing^1^. So, the future research of stroke must expand its horizon to find new strategies. Neurogenesis is a promising step toward the recovery and restoration of brain function after stroke and other neurogenerative diseases^39–41^. Our study demonstrated that in an early stage of ischemic stroke, IL-17, as an important indicator of poor prognosis, was involved in the proliferation relevant signal pathway-PI3K/Akt pathway, and responsible for suppressing local NSCs stemness and neurogenesis, as well as infraction area. The pro-neurogenesis and cognitive beneficial effect induced by administering As-IV was observed, accompanied with anti-apoptosis and pro-proliferation effect. IL-17 and As-IV did not work on cell apoptosis and proliferation independently completely, but interactively. IL-17 expression was decreased significantly by giving As-IV. Similarly, NSCs’ proliferation ability was promoted by As-IV but inhibited by IL-17A in vitro, with Akt/GSK-3β and Wnt/β-catenin pathway regulated.

Neuroinflammation participates in almost every step of ischemic brain repair, including neurogenesis^42^. Microglia are resident cells that regulate brain development, maintenance of neuronal networks, and injury repair. Microglia serve as brain macrophages but they are distinct from other tissue macrophages owing to their unique homeostatic phenotype and tight regulation by CNS microenvironment. They are responsible for eliminating microbes, dead cells, redundant synapses, protein aggregates and soluble antigens which are endangerous to the CNS. Furthermore, microglia are pivotal mediators of neuroinflammation and can induce or modulate a broad spectrum of cellular responses. Under different stimulations, microglia may polarize into either of two phenotypes that conduct drastically different pathophysiological behaviors^43^. Microglia activation towards M1 phenotype augments post-stroke neuroinflammation and decreases neurogenesis^44,45^ by secreting pro‐inflammatory factors IL‐1, IL‐6, IL‐12, and nitric oxide. While the M2 polarized microglia support neurogenesis by promoting neuron proliferation and secreting anti-inflammatory factors, as well as producing specific trophic factors that increase neural precursor cell proliferation and neuroblast migration^42^. So we first observed the M2 polarized microglia by signing Iba-1 protein, with immunohistochemistry staining (Fig. 1b), suggesting M2 polarized microglia appear fairly late in acute ischemic cortex. The apoptosis induced by ischemic stroke has been well studied^46–48^, so we did not assess it very thoroughly, only by observing Caspase 3 protein expression and TUNEL positive neurons (Figs. 6, 7).

Neurogenesis is the generation of new neurons in the brain that occurs by the division of NSCs and subsequent maturation in NPCs and then into neurons^49^. Traditionally, neurogenesis requires multiple steps, including NPCs or NSCs mobilization, proliferation, neuronal migration, neuron maturation, and synaptic reconstruction. Understandably neurogenesis plays a crucial role during embryonic development for the construction of the nervous system but it is now clearly established that it continues even during adult life in some region of the mammalian brain. The main well-documented neurogenic areas of the adult brain are the subgranular zone (SGZ) of the hippocampus DG, where new granule cells originate, and the SVZ of the lateral ventricles where new olfactory bulb interneurons are generated. Unlike during embryonic development, neurogenesis proceeds in the adult brain at a slow rate since adult NSC are mainly quiescent. The quiescent NSCs are always activated by some injury, like ischemia. Resident astrocyte‐derived neurons could transdifferentiate into morphologically mature and functional neurons, with typical neuronal morphology and electrophysiological activity. It has been shown that striatal astrocytes possess the ability to either produce neuroblasts or acquire NSC‐like properties by expressing NSC related protein such as Nestin, Sox2, and DCX after stroke^50^. In this study, in ischemic cortex, GFAP was highly coincident with Nestin or Sox2, which indicates the possibility of resident astrocyte‐derived neurons in adult cortex (Figs. 3, 5). We think these resident astrocyte‐derived neurons can grow into immature neurons, even mature neurons marked by double positive BrdU and NeuN (Fig. 4c)

In the present study, to observe the neurogenesis in ischemic cortex after stroke regulated by IL-17 and As-IV, NPC markers-Nestin, GFAP and Sox2 and immature neuron marker-DCX, and mature neuron marker-NeuN protein expression was chose (Figs. 3–5**)**. PI3K/Akt/GSK-3β is a well-known classical pathway to regulate both cell proliferation and apoptosis^51–53^. In our RNA-seq results, the DEGs were also enriched in PI3K/Akt pathway by knocking out IL-17 (Fig. 2). Beside, Wnt are produced by NSCs and astrocytes in both V-SVZ and SGZ, where NSCs respond to canonical Wnt signaling to promote NSC self-renewal and NPC proliferation^54^. In our previous study, we found that in the ischemic hippocampus, neurogenesis in hippocampal DG was activated, accompanied with Wnt/β-catenin pathway upregulated. So, to conform the mechanism for neurogenesis, the PI3K/Akt and Wnt/β-catenin pathway were assessed at the same time in this study (Figs. 2, 6–8**)**.

IL-17 is the most widely investigated cytokine of this family and IL-17 is mainly produced by γδ T cells in the acute phase of stroke^55^. Recent studies show that Th17 cells and their signature cytokine IL-17 also has a role in a wide variety of neurological diseases, like ischemic brain injury^4^. Our and other previous studies have shown that IL-17, whose expression peaked on day 3 after stroke contributed to acute ischemic brain injury^17^ (Fig. 1 a, b). Upregulating IL-17 expression indicates a poorer treatment effect and prognosis^5^ and decreased IL-17 is relative to improved motor function in mice^6,7^. Administration of IL-17A can nullify the microglial M2 polarization to inhibit anti-inflammation activity^13^. In this study, we found IL-17’s negative effect on ischemia might derive from the inhibition of anti-inflammation by secreted IL-17 to suppress the elevated Iba-1 protein expression (Fig. 1b). In this study, in order to find out IL-17’s exact function, we analyzed the transcriptome sequencing in ischemic cortex between WT and IL-17 KO mice, and the results showed that DEGs were enriched in some pathway, like cancer, TNF, PI3K/Akt, p53 and IL-17 pathway (Fig. 1 d, e). Then we chose PI3K/Akt pathway as the key pathway to find the underlying mechanism of IL-17’ s effect on ischemic stroke. For observing the different development stage of neurogenesis, NPCs, immature neuron and mature neuron markers were immunohistochemistry stained, respectively (Fig. 8d). A significant increase of Nestin^+^, Sox2^+^, and GFAP^+^ cells numbers at 3 dpi and BrdU^+^-DCX^+^ cells numbers at 7 dpi were found around the ischemic cortex and increased by knocking out IL-17 (Figs. 3, 4).

As-IV makes potential neuroprotective effect in experimental models of Parkinson’s disease, Alzheimer’s disease and cerebral ischemia, by antioxidant systems, reducing inflammation and oxidative stress^22^. As-IV could also be a new therapeutic drug candidate for post-stroke treatment to effectively promote NSCs proliferation and neurogenesis in transient cerebral ischemic brains^28^. We found that mice treated with As-IV had greater double positive cell numbers of Nestin/GFAP and Sox2/GFAP in the ischemic infarction cortex compared with control mice, with increasing BrdU^+^-DCX^+^/BrdU^+^ cells percentage **(**Fig. 5a–e**)**. As-IV may ameliorate immobilized stress-induced anxiety and inflammation^56^. To access anxiety-like behavior, open field test and elevated plus maze test was used. These data demonstrated that the anxiety-like behavior appeared after ischemic stroke and relieved after administering As-IV **(**Fig. 5f–i**)**. Besides, As-IV reduces the release of pro-apoptotic proteins and resultantly protects neurons from apoptosis and parthanatos^23^. In this study, at 3 dpi, there were significantly less infarction area, numbers of TUNEL and NeuN double positive cells, and level of Caspase 3 protein expression in the cortex of As-IV treated mice (Fig. 6a, c), which indicated that As-IV reduced the neural apoptosis. Members of the Caspase family of proteases play essential roles in the initiation and execution of apoptosis. These Caspases are divided into two groups: the initiator Caspases (Caspase-2, -8, -9, and -10), which are the first to be activated in response to a signal, and the executioner Caspases (Caspase-3, -6, and -7) that carry out the demolition phase of apoptosis^36^. So we only chose Caspase 3 expression to observe the apoptosis.

GSK-3β is considered a key player to regulate neurogenesis and synaptic function via PI3K/Akt signaling pathway^57,58^ and inhibition of GSK-3β increases the proliferation of neural progenitors by upregulating Wnt signaling in mouse^18,20^. Some research showed that As-IV evoked Akt phosphorylation, and subsequent induced phosphorylation of GSK-3β at Ser9 (that is, inactivation) to promote neurite outgrowth in vitro^59^. However, in some cancer models, As-IV inhibits the Akt/GSK-3β/β-catenin signaling axis by decreasing the phosphorylated forms of Akt and GSK-3β^26,27^. The controversial opinion on As-IV’s effect on Akt/GSK-3β/β-catenin signaling pathway still exists, so we ought to find the exact mechanism of As-IV’s neuroprotection. Then, in our study, the expression of phosphorylated forms of PI3K and Akt was upregulated by As-IV (Fig. 6d).

Therefore, IL-17 and As-IV exerts opposite effect on neurogenesis after stroke. However, they did not work independently, but interactively. IL-17 expression was downregulated significantly by administering As-IV both in vivo and in vitro (Figs. 6c, 7c, 8b). So, we considered IL-17 as a key target for As-IV’s neuroprotection and explored the underlying mechanism further. In vivo, neural apoptosis was increased by knocking out IL-17 and decreased by administering As-IV (Fig. 7a, c, e). Wnt2, p-GSK-3β, GSK-3β and β-catenin protein expression was also upregulated by either knocking out IL-17 or administering As-IV (Fig. 7b, d, f), which is consistent with some research^8,60,61^. In vitro, As-IV could make positive effect on promoting proliferation and differentiation of NSCs^62,63^. For this study, we found the effects of 20 μM As-IV made protective effect on sphere formation and adherent culture cells proliferation and differentiation, while recombinant mouse IL-17A inhibited NSCs’ stemness (Fig. 8a). To understand the underlying mechanism in cultured NSCs, Akt/GSK-3β pathway and Wnt/β-catenin pathway protein expression was observed by western blots (Fig. 8b). The results showed that p-Akt, p-GSK-3β, Wnt2, and β-catenin protein expression was increased by administering As-IV, while decreased by giving IL-17A (Fig. 8c).

In conclusion, ischemic stroke remains a leading cause of adult disability and further research is still needed. First, *Il-17* mRNA and IL-17 protein expression was significantly increased and peaked at 3 dpi after stroke. Knocking out IL-17 contributed to upregulating PI3K/Akt pathway, activating NSCs’ stemness and promoting neurogenesis after ischemic stroke, but with increasing infarction area. Moreover, the treatment of stroke mice with As-IV was helpful for inhibiting neural apoptosis, promoting the neurogenesis and eventually relieving the cognitive deficits after stroke. To find out how As-IV works on IL-17, western blots were assessed. We found that cell apoptosis was decreased by As-IV and cell proliferation was activated by As-IV. For the mechanism, knocking out IL-17 and administering As-IV exerts similar promotion effect on cell proliferation via Wnt/β-catenin pathway. Similarly, NSCs’ proliferation ability was promoted by As-IV but inhibited by IL-17A in vitro, with Akt/GSK-3β and Wnt/β-catenin pathway regulated. Thus, IL-17 is a key effector of As-IV and knocking out IL-17 might exert protective effect on promoting neurogenesis, with Akt/GSK-3β and Wnt/β-catenin pathway upregulated.

## Materials and methods

### Animals

This study complied with the ARRIVE guidelines. All procedures were conformed to the Guide for the Care and Use of Laboratory Animals published by the National Institutes of Health (NIH) and approved by the Institutional Animal Care and Use Committee of Air Force Medical University (Certification No. IACUC-20180905). All WT and IL-17 knock out (KO) mice, aged 4–8 weeks old, were housed in a room maintained at a constant temperature and on a 12-h light/dark cycle (light from 08:00 to 20:00) and they can get water and food at will. The IL-17 KO mice had a pure C57BL/6 background. All experiments were performed in age-matched mixed gender mice by experimenters blinded to the genotypes and groups. The sample size was estimated about 130 mice before the experiment, including five pregnant mice, 93 WT mice and 32 IL-17 KO mice. If the mouse was dead during the operation, the mouse would be excluded from the analysis. All mice were allocated randomly to different experimental groups.

### Photochemical brain ischemia model and mouse treatment

Focal cortical ischemia was induced by photothrombosis of the cortical microvessels as described previously^64,65^. Rose bengal (Sigma, Cat# 330000) was injected ip. at a concentration of 100 mg/kg^65^. Then, a skull window was carefully made 0.3–2.3 mm posterior to the Bregma and 0.5–3.0 mm right of the midline without injuring the brain tissue. The brain was illuminated for 15 min by using a cold light source (Zeiss FL1500 LCD) of the appropriate intensity for 15 min after the rose bengal injection. To observe neurogenesis in the hippocampus, S-phase marker 5-bromo-2′-deoxyuridine (BrdU), was injected ip. at a dose of 50 mg/kg once per day beginning on the second day after stroke and continuing for 6 days^66,67^. For the As-IV groups, 200 mg/kg As-IV (Macklin, Cat# A800922) was injected intravenously (iv.) via the tail vein for three consecutive days beginning on the stroke day^68^.

### Western blotting

Ischemic cortex tissue samples were extracted at 3 dpi and homogenized in RIPA lysis buffer. After SDS-PAGE and protein transfer, membranes were incubated with primary antibodies including rabbit anti-IL-17 (1: 1 000, Abcam, Cat# ab79056), mouse anti-Wnt2 (1: 5 000, Abcam, Cat#66656-1-lg), rabbit anti-p-PI3K p85 ((Tyr458)/p55 (Tyr199), 1:1 000, Cell signaling, Cat#17366), rabbit anti-PI3K p-85(1:1 000, Cell signaling, Cat#4257), rabbit anti-p-Akt (Ser473, 1:1 000, Cell signaling, Cat#4060), rabbit anti-Akt (1:1 000, Cell signaling, Cat#4685), rabbit anti-Caspase 3 (1:1 000, Cell signaling, Cat#14220), rabbit anti-β-catenin (1:1 000, Cell signaling, Cat#25362), rabbit anti-p-GSK-3β (Ser9, 1:1 000, Cell signaling, Cat#5558), rabbit anti-GSK-3β (1:1 000, Cell signaling, Cat#12456), and rabbit anti-β-actin (1:3 000, Cell signaling, Cat#5125) overnight at 4 °C, followed by incubation with HRP-conjugated anti-rabbit or anti-mouse IgG (1: 5 000, Proteintech, USA) for 3 h at room temperature. Bands were visualized with an ECL kit (Thermo).

### Transcriptome sequencing

mRNA in the cortex of WT and IL-17 KO mice was extracted with ESscience RNA-Quick Purification Kit (YiShan Biotech, Shanghai, China). Then the library construction and RNA-sequencing (RNA-seq) were performed at Shanghai Sinomics Corporation (Shanghai, China) with Illumina NovaSeq 6000 (Illumina, USA), followed by the computational analysis they provided. The criteria for differential genes was set up with *P* value<0.01 and fold change >1.5 or <0.5. Differential expression genes (DEGs) analysis for mRNA was performed using R package edge R. DEGs with |log2(FC)| value >1 and *q* value < 0.05, considered as significantly modulated, were retained for further analysis.

### Real-time reverse transcription PCR (RT-PCR)

Total RNA was extracted from dissected cortical tissues at 1, 3, 5, and 7 dpi using the PerfectPure RNA cell kit (5 Prime, Fisher) and quantified by spectrometry at 260 and 280 nm. The RNA was reverse-transcribed using the High-Capacity cDNA Archive kit (Applied Biosystems) and real-time PCR with cDNA was performed using the ABI 7500 Fast System, according to the manufacturer’s protocol (Applied Biosystems). mRNA levels of target genes, including *Il-17a, Akt*, and *Gsk-3β* qRT-PCR primer set, were normalized to the mRNA levels of *Gapdh* using the ΔΔCt method. Primers for quantitative PCR: *Gapdh*-Fw: 5′-TGGTGAAGGTCGGTGTGAAC-3′; *Gapdh*-Rv: 5′-GCTCCTGGAAGATGGTGATGG-3′; *Il-17a*-Fw: 5′-CAGACTACCTCAACCGTTCCA-3′; *Il-17a*-Rv: 5′-CTGAGCTTCCCAGATCACAGA-3′; *Akt-*Fw: 5′-GGGGCAGAAAAGCAATAATGT-3′; *Akt*-Rv: 5′-CATCCATAGGGTGAGGACAGTT-3′; *Gsk-3β-*Fw: 5′-TCCGACTGCGGTATTTCTTCTA-3′; *Gsk-3β*-Rv: 5′-ATCACAGGGAGTGTCTGCTTGG-3′.

### Immunohistochemistry and TUNEL staining

Slides were blocked in 0.01 M PBS containing 0.3% Triton X-100 and 3% bovine serum albumin (BSA) for 1 h, and incubated with primary antibodies overnight at room temperature. The primary antibodies used were as follows: rabbit anti-IL-17 (1:500, Abcam, Cambridge, UK, Cat# ab79056), goat anti-Iba-1 (1:600, Abcam, Cambridge, UK, Cat# ab5076), rabbit anti-NeuN (1:600, Millipore, USA, Cat#ABN78), goat anti-Nestin (1:500, Santa Cruz, Delaware, Cat# sc-21249), goat anti-Sox2 (1:50, Santa Cruz, Delaware, Cat# sc-365823), guinea pig anti-DCX (1:600, Millipore, USA, Cat# ab2253), rabbit anti-GFAP (1:1 000, DAKO, Denmark, Cat# z0334), rabbit anti-Caspase 3 (1:600, Cell signaling, Cat#14220), and mouse anti-Wnt2 (1:800, Abcam, Cambridge, UK, Cat#66656–1-lg). Corresponding secondary antibodies conjugated with Alexa Fluor 594 (donkey anti-guinea pig Cat# 706-585-148, or anti-rabbit Cat# 711-585-152, IgG, 1:800, Jackson ImmunoResearch), and Alexa Fluor 488 (anti-goat Cat# 705-545-147, or anti-mouse Cat#115-005-205, IgG, 1:500, Jackson ImmunoResearch) were incubated with the sections for 2–4 h at room temperature protected from light. The nuclei were counterstained with Hoechst 33342 (1:3 000, Sigma, St. Louis) for 15 min.

Brain slices were immersed in 2 N HCl for 20 min at 37 °C to denature DNA and washed by Borate buffer (0.1 M) and PBS. After permeabilized, the slices were incubated with primary antibody, rat anti-BrdU (1: 200, Abcam, Cambridge, UK, Cat# ab6326), overnight at room temperature and incubated with corresponding secondary antibody conjugated with Alexa 488 (donkey anti-rat Cat# 712-545-153) for 2–4 h at room temperature.

For TUNEL/NeuN double-staining, immunostaining of NeuN was performed first, and then TUNEL staining followed by according to the manual of DeadEND^TM^ TUNEL system (Promega)^69^.

### Open field test

The open field test was carried out in a white opaque plastic chamber (50 × 50 × 35 cm) at 3 dpi as described^70^. The open field was divided into 16 squares with same area. The central four squares in were defined as central area, and the remaining as periphery area. For each test, mouse was gently placed in one corner, and the movement was recorded for 10 min with a video tracking system. The time spent and distance traveled in the central area and the total distance traveled in the field were measured using the SMART software (SMART 3.0, Panlab S.L.U.). Between each test, 75% ethanol was used to clean the open field area.

### Elevated plus maze test

The elevated plus maze test was performed at 7 dpi and the maze was placed 50 cm above the floor and consisted of two open arms and two closed arms (30 × 5 cm and 15 cm wall height for the closed arms)^70^. Each mouse was placed onto the center area, heading toward the same open arm, and videotaped in the following 5 min. The time spent and moving distance in the open arms, and the total movements in both open and closed arms were analyzed using the software SMART 3.0. The maze was cleaned by 75% ethanol between tests.

### Primary cell culture and treatments

The cortex, removed from E12-E14 mouse embryos, was dissected and digested in 0.125% trypsin for 10 min at 37 °C^71^. NSCs were cultured in Neurobasal medium supplemented with 2% B27, 1% N2, 2% Gln, 20 ng/ml recombinant murine epidermal growth factor (EGF, Peprotech, Cat# 315-09), and 20 ng/ml recombinant murine fibroblast growth-basic factor (FGF, Peprotech, Cat# 450-33) for 7–10 days^72^. To observe NSCs proliferation under different conditions, recombinant mouse IL-17A (IL-17A, Novoprotein, Shanghai, China, Cat# CX14) 1 ng/ml, and 20μmM As-IV were used, according to the product introduction^73^. The morphology and growth feature of the neural spheres and adherent culture NSCs were observed and taken pictures by inverted microscope after 3 days’ treatment, and collected for western blots.

### Image collection and statistical analysis

All images of immunofluorescence staining were acquired with Olympus FV1000 and all viable cells were pictured by inverted microscope. Images were analyzed by Imaris software. Data were evaluated by one-way ANOVA and the Dunnett’s multiple-comparison test using the InStat program (GraphPad). Where appropriate (comparison of two groups only), two-tailed unpaired t-tests were performed. Data shown are representative of three or more independent experiments. Cell counting and quantification were performed by an investigator who was blinded to the experimental design. Data are presented as the mean ± standard error. *P*<0.05 was taken as the level of significance.

## Data Availability

Datasets are available on request and the raw data supporting the conclusions of this manuscript will be made available by the authors, without undue reservation, to any qualified researcher. The transcriptome sequencing results can be found in NCBI https://www.ncbi.nlm.nih.gov/sra/PRJNA641783. It will be released on 2020-07-31.
